# In Vivo Characterization and Application of the PHA Synthase from *Azotobacter vinelandii* for the Biosynthesis of Polyhydroxyalkanoate Containing 4-Hydroxybutyrate

**DOI:** 10.3390/polym13101576

**Published:** 2021-05-14

**Authors:** Pei-Shze Mok, Jo-Ann Chuah, Nazalan Najimudin, Pauline-Woan-Ying Liew, Bor-Chyan Jong, Kumar Sudesh

**Affiliations:** 1 School of Biological Sciences, Universiti Sains Malaysia, Gelugor 11800, Malaysia; pepsimok@hotmail.com (P.-S.M.); jannchuah@gmail.com (J.-A.C.); nazalan@usm.my (N.N.); 2 Agrotechnology and Bioscience Division, Malaysian Nuclear Agency, Bangi 43000, Malaysia; paulineliew@nuclearmalaysia.gov.my (P.-W.-Y.L.); jongbc@nuclearmalaysia.gov.my (B.-C.J.)

**Keywords:** polyhydroxyalkanoate (PHA), *Azotobacter vinelandii*, P(3HB-*co*-4HB), PHA synthase, substrate specificity, S-layer

## Abstract

Polyhydroxyalkanoate (PHA) is a biodegradable thermoplastic naturally synthesized by many microorganisms, and the PHA synthase (PhaC) is known to be the key enzyme involved in determining the material properties and monomer composition of the produced PHA. The ability to exploit widely distributed, commonly found soil microorganisms such as *Azotobacter vinelandii* to synthesize PHA containing the lipase-degradable 4-hydroxybutyrate (4HB) monomer will allow for convenient production of biocompatible and flexible PHA. Comparisons between the *A. vinelandii* wild type and mutant strains, with and without a surface layer (S-layer), respectively, in terms of gene or amino acid sequences, synthase activity, granule morphology, and PHA productivity, revealed that the S-layer is the sole factor affecting PHA biosynthesis by *A. vinelandii*. Based on PHA biosynthesis using different carbon sources, the PhaC of *A*. *vinelandii* showed specificity for short-chain-length PHA monomers, making it a member of the Class I PHA synthases. In addition, it was proven that the PhaC of *A*. *vinelandii* has the inherent ability to polymerize 4-hydroxybutyrate (4HB) and the mediated accumulation of PHA with 4HB fractions ranging from 10 mol% to as high as 22 mol%. The synthesis of biocompatible PHA containing tailorable amounts of 4HB with an expanded range of elasticity and lipase-degradability will enable a wider range of applications in the biomedical field.

## 1. Introduction

The demand for synthetic plastics has increased as the human population increases. The widespread usage of petroleum-based synthetic plastic in various applications such as mobile and transport, construction and building, as well as entertainment activities, has created severe environmental pollution and depletion of petroleum. Limitations in resources needed for the synthesis of plastics triggered many researchers to develop a new type of plastic. Polyhydroxyalkanoate (PHA) was found to have similar properties to synthetic plastics such as polypropylene (PP) and hence could be a potential candidate to replace certain types of petroleum-based synthetic plastics. Unlike chemically synthesized plastic, PHA is biosynthesized by microbial cells under nutrient imbalance. The PHA granules are stored in the body of microbial cells as carbon and energy reserves for when the surrounding environment lacks a carbon source for cell survival [1,2,3,4].

PHA synthase (PhaC) is the key enzyme that plays the central catalytic role in PHA production. PhaC uses coenzyme A (CoA) thioesters of hydroxyalkanoic acids (HAs) as substrates and catalyzes the polymerization of HAs to yield PHA with a concomitant release of CoA. Hence, PhaC has a dominant function in determining the general properties of produced PHA, such as molecular weight, monomer composition, and polydispersity. Many studies were carried out to characterize PhaCs from various environments [5,6].

Among the different polymers that were discovered, poly(3-hydroxybutyrate) (P(3HB)) is the first and most studied PHA. However, P(3HB) is known to have poor properties such as brittleness and a narrow thermal processing window, making its application difficult. Incorporation of other monomers such as 4-hydroxybutyrate (4HB), which is biocompatible and lipase-degradable, can improve the properties of the resultant PHA copolymer and enable its application in the medical field [7,8]. By adjusting the monomer composition of the copolymer, its crystallinity and elasticity can be altered. PHA can also serve as biomaterial for use by the aging population [9]. Depending on the requirements of a target application, different copolymers could be synthesized using microorganisms that can utilize more than one type of monomer.

*Azotobacter vinelandii* is a commonly found gram-negative, non-pathogenic, and PHA-producing soil bacterium that can fix atmospheric nitrogen and generate different products such as PHA, alginate, and plant hormones [10]. This bacterium was reported to produce PHA consisting of only 3HB and 3-hydroxyvalerate (3HV) [11]. According to previous studies, *A*. *vinelandii* UWD could synthesize around 2.5 and 6.8 g/L of P(3HB) using 20 g/L of glucose and sugar beet molasses, respectively [12]. Besides, *A*. *vinelandii* UWD and *A*. *vinelandii* mutant OPNA strains could synthesize around 25 g/L of P(3HB) through fed batch cultivation [13,14]. By using glucose and precursors such as propionate, heptanoate, nonanoate, or trans-2-pentenoate, *A*. *vinelandii* UWD could synthesize poly(3-hydroxybutyrate-*co*-3-hydroxyvalerate) (P(3HB-*co*-3HV)) [11]. According to Mok and colleagues, the *A. vinelandii* Lipman ATCC 12837 wild type strain has a surface layer (S-layer) surrounding the cells that allows the bacterium to attach to the plant root easily. However, a mutant strain of *A. vinelandii* (Δ*Avin_16040*) without the S-layer was found to produce higher amounts of PHA compared to the wild type strain under the same conditions, suggesting that the mutant strain without the S-layer could consume the carbon source much easier compared to the wild type strain [15].

In this study, the incorporation of other monomers, especially 4HB by the *A. vinelandii* wild type and mutant strains, was evaluated by providing structurally-related carbon sources during cell cultivation for PHA biosynthesis. The PHA synthase gene was also characterized by its expression in PHA-negative, *Cupriavidus necator* mutant host strains. If the polymerization of other monomers in addition to 3HB is possible using the synthase of *A. vinelandii*, it could be a practical tool for the production of different types of highly useful PHA as this bacterium can be easily found in the soil.

## 2. Materials and Methods

### 2.1. Bacterial Strains and Cultivation Conditions

All PHA production was carried out using the *A. vinelandii* Lipman ATCC 12837 wild type strain, *A. vinelandii* Δ*Avin_16040* mutant strain, and *C*. *necator* PHB^−^4 and Re2058 strains containing PHA synthase from *A. vinelandii* (Table 1). The *A. vinelandii* Δ*Avin_16040* mutant strain without the surface layer (S-layer) producing gene was constructed so that the attachment of the bacterial cell towards the plant could be reduced [16]. Homologous replacement was carried out to delete the *Avin_16040* gene that produced the S-layer protein.

For short-term storage, *A. vinelandii* strains were cultivated at 30 °C on Burk’s medium (BM) agar supplemented with 20 g/L of sucrose, while *C*. *necator* strains harboring the PHA synthase from *A. vinelandii* were cultivated on nutrient rich (NR) agar supplemented with 50 µg/L of kanamycin at the same temperature. For long-term storage, stocks of respective bacterial strains were prepared with glycerol (final concentration of 20% *v/v*) and kept at −80 °C.

### 2.2. Biosynthesis of PHA

For the *A*. *vinelandii* wild type strain and *A*. *vinelandii* Δ*Avin_16040* mutant strain, cell cultivation was carried out, as described previously [15]. The bacterial cells were first streaked on NR agar plates consisting of 10 g/L of peptone, 2 g/L of yeast extract, 10 g/L of meat extract, 5 g/L of glucose, and 15 g/L of agar powder followed by incubation at 30 °C for 2 days. Sugar was added in the NR medium to increase the cell biomass [15]. Cells (3 loopfuls) were added into 50 mL of NR broth (excluding glucose and agar powder) containing 20 g/L of sucrose and cultivated at the same temperature with an agitation speed of 200 rpm for 15 h to obtain sufficient cell biomass for subsequent steps. The cell culture (3% *v/v*) was transferred aseptically into 50 mL of minimal medium for PHA accumulation (MMPHA) at pH 7 along with the addition of 30 g/L of fructose as a carbon source, 0.54 g/L of urea as a nitrogen source, and other nutrients, as described in previous studies [15,21]. The compositions of MMPHA broth and other nutrients were 4.6 g/L of di-sodium hydrogen phosphate, 4 g/L of sodium dihydrogen phosphate, 0.45 g/L of potassium sulfate, 0.062 g/L of calcium chloride, 0.39 g/L of magnesium sulfate, and 1 mL/L of trace elements (TE) solution. The compositions of TE solution were 15 g/L of iron(II) sulfate, 2.4 g/L of manganese sulfate, 2.4 g/L of zinc sulfate, and 0.48 g/L of copper(II) sulfate in 0.1 M of hydrochloric acid solution. The cells were then cultured at the same temperature and agitation speed for 72 h for PHA production, and then harvested by centrifugation at 4 °C, 8590 *g* for 10 min. The harvested cells were frozen and freeze-dried using a freeze dryer (LABCONCO FreeZone, Kansas City, MO, USA).

For *C*. *necator* PHB¯4 and *C*. *necator* Re2058 transformants harboring the PHA synthase gene from *A*. *vinelandii*, the cells were streaked on a NR agar plate (without glucose) supplemented with 50 µg/mL of kanamycin and cultivated under similar conditions as *A*. *vinelandii* cells by adding 50 µg/mL of kanamycin in respective media for cell growth and PHA accumulation. After cultivation, the cells were harvested and freeze-dried under similar conditions as the *A*. *vinelandii* strains.

Different carbon sources including glucose, fructose, sucrose, crude palm kernel oil (CPKO), molasses, and glycerol were screened during cultivation of the transformed cells to obtain higher amounts of dry cell and PHA. Precursors or structurally related carbon sources including sodium valerate, sodium 4-hydroxybutyrate (Na-4HB), sodium 5-hydroxyvalerate (Na-5HV), 1,4-butanediol (1,4-BD), gamma-butyrolactone (γ-BL), 4-methylvaleric acid, sodium propionate, sodium hexanoate, sodium heptanoate, sodium octanoate, and sodium nonanoate were also added to screen the ability of the *A. vinelandii* ATCC 12837 wild type strain, Δ*Avin_16040* mutant strain, and transconjugants of *Cupriavidus necator* to incorporate different monomers for PHA production.

### 2.3. In Vitro PHA Synthase Activity Assay

Reagents including a 200 mM Tris-HCl buffer (pH 7.8), 50 mM stock solution of (*R*)-3-hydroxybutyryl Coenzyme A (3HB-CoA), and 4 mM of 5,5′-dithio-bis-(2-nitrobenzoic acid) (DTNB) reaction solution were prepared to evaluate the activity of PHA synthase. In order to plot a standard curve of absorbance against a concentration of CoA, different concentrations of CoA (0 to 0.3 mM) were dissolved in a total volume of 800 µL DTNB reaction mixture and Tris-HCl buffer. The absorbance of the solution was read using a spectrophotometer at a wavelength of 412 nm. For sample preparation, the bacterial cells were harvested under centrifugation (4 °C, 8590 g for 10 min) and washed using distilled water after 24 h of cultivation in MMPHA broth. The washed cells were resuspended in 2 mL of Tris-HCl buffer (pH 7.8) and lysed using a pressure cell press (Thermo IEC, Rockford, IL, USA) at 1000 psi. The crude extract after pressing was centrifuged at 4 °C and 10738 g for 10 min. Both the supernatant and pellet were kept, and the protein concentrations for both samples were measured using the Bradford protein quantification method [22]. A total volume of 800 µL solution was made up by a combination of 40 µg of protein from the supernatant or pellet and solutions with a final concentration of 0.15 mM of 3HB-CoA, 0.15 mM of DTNB, 100 mM of Tris-HCl buffer (pH 7.8), and distilled water. The absorbance of the solution was read every 30 s in a spectrophotometer at a wavelength of 412 nm. The concentration of CoA released was determined based on the standard curve, and the total PHA synthase activity was calculated. One unit of enzyme (U) was defined as the amount of enzyme required to release 1 µmol of CoA from 3HB-CoA per minute.

### 2.4. Comparison of phaC Sequences between A. vinelandii ATCC 12837 Wild Type Strain and ΔAvin_16040 Mutant Strain

The sequences of *phaC* from both the *A. vinelandii* ATCC 12837 wild type and Δ*Avin_16040* mutant strains were amplified using specifically designed primers Av_PhaC-F: 5′-ATGGATCAAGCCCCCTCTTTC-3′ and Av_PhaC-R: 5′-TCAGCCTTTCACGTAACG GCC-3′ through a colony polymerase chain reaction (PCR) as described in a previous study [23]. The raw sequencing results were then trimmed using a computer software (DNA Baser) and assembled using a nucleotide basic local alignment search tool (BLAST) in the National Center for Biotechnology Information (NCBI).

### 2.5. Expression of PHA Synthase Gene (phaC) from A. vinelandii ΔAvin_16040 Mutant Strain in C. necator PHB¯4 and Re2058

The plasmids pBBR1MCS-2 and pCB113 were used as vectors for gene expression in *C*. *necator* PHB^−^4 and Re2058. For pBBR1MCS-2, a pair of primers was designed with underlined restriction sites of *Hind*III and *Xho*I as follows: *Xho*I_AvPhaC-F (5′-AATCTCGAGCAAAGGAGGATCATTTCAATGGATCAAGCCCCCTCTTTCACA-3′) and *Hind*III_AvPhaC-R (5′-AGTAAGCTTTCAGCCTTTCACGTAACGGCC-3′). These primers were constructed to amplify *phaC* of the *A*. *vinelandii* Δ*Avin_16040* mutant strain together with restriction enzymes. The amplified *phaC* and pBBR1MCS-2 were then digested separately with *Hind*III and *Xho*I. Both digested *phaC* and pBBR1MCS-2 were ligated using a DNA Ligation Kit (Takara Bio Inc., Shiga, Japan), resulting in pBBR1MCS2-*pha*C*_Av_* that indicated the insertion of *phaC* from *A*. *vinelandii* into pBBR1MCS-2. This ligated product was transformed into commercial *E*. *cloni* 10G chemically competent cells (Lucigen, Middleton, WI, USA) to ensure the ligation efficiency and cultivated on the Luria Bertani (LB) agar containing 50 µg/mL of kanamycin. The transformation was conducted according to the manufacturer’s protocol. After that, the plasmid was extracted from the colonies growing on the NR agar plate supplemented with kanamycin and transferred into *Escherichia coli* S17-1. The presence of pBBR1MCS2-*pha*C*_Av_* in the transformed cells was verified by sequencing. According to Friedrich and colleagues, transconjugation was carried out between the transformed *E*. *coli* and *C*. *necator* PHB¯4 by mixing the cells at a 1:1 ratio and cultivating for 8 h in NR agar at 30 °C [24]. After 8 h of incubation, the cells were resuspended using NR broth and spread on Simmons’ citrate agar supplemented with 300 µg/mL of kanamycin. The agar plate was incubated at 30 °C for two days, and the color of agar changed from green to blue when the cell started to utilize citrate for growing. The cell colonies were selected and proceeded with PCR using plasmid-specific primers for verification. The sequences of the primers were SeqPri_CnPro F (5′-GCGTCTCCATGCGAGAATGTC-3′) and R_pBBR1MCS2_1751 (5′-CACACAGGAAACAGCTATGAC-3′).

The steps to transfer *phaC* from the *A*. *vinelandii* Δ*Avin_16040* mutant strain into *C*. *necator* Re2058 were similar to that for the transfer of *phaC* from the *A*. *vinelandii* Δ*Avin_16040* mutant strain into *C*. *necator* PHB¯4 by replacing pBBR1MCS-2 with pCB113 and restriction enzymes of *Xho*I and *Hind*III to *Swa*I. The primers used to clone *phaC* from the *A*. *vinelandii* Δ*Avin_16040* mutant strain into pCB113 were *Swa*I_*Av*PhaC_F (5′-ATCATTTAAATAGGAGGAGGCGCATGGATCAAGCCCCCT-3′) and *Swa*I_*Av*PhaC_R (5′-AGCATTTAAATTCAGCCTTTCACGTAACGGCCT-3′) with the restriction sites of *Swa*I underlined. The plasmid-specific primers were SwaI_AvPhaC_F and R_phaA_157 (5′-TGATGACTTCGCTCACCTGCTC-3′). Glycerol stocks of the cells were then prepared with 20% *v/v* of sterile glycerol and kept in a −80 °C freezer.

### 2.6. Gas Chromatography (GC) Analysis

The PHA content and monomer composition of the freeze-dried cells were determined by GC analysis [25]. The dry cells (15 to 20 mg) were weighed in a screw-capped test tube while 2 mL of chloroform and 2 mL of methanolysis solution (85% *v/v* of methanol and 15% *v/v* of sulfuric acid) were added into the tubes, followed by heating at 100 °C for 140 min. The tubes were occasionally tapped throughout the heating process to homogenize the sample. The samples were then left to cool to room temperature (around 25 °C). Two layers of solution were formed after adding 1 mL of distilled water and vortexing. The lower layer (hydroxyacyl methyl esters) was transferred into a tube containing sodium sulfate powder and shaken to remove trace amounts of water. A one to one ratio of hydroxyacyl methyl ester solution and 0.2% *v/v* of caprylic acid methyl ester (CME) solution with a total volume of 1 mL was mixed in a vial. CME solution was used as an internal standard. The sample was then analyzed in a gas chromatograph (Shimadzu, Kyoto, Japan) equipped with an AOC-20i Auto injector (Shimadzu, Kyoto, Japan), SPB-1 Capillary GC column and a flame ionization detector (FID). The carrier gas used was nitrogen.

### 2.7. Nuclear Magnetic Resonance (NMR) Analysis

Proton NMR analysis was carried out to confirm the monomer composition of PHA using a Bruker Advance 500 spectrometer (Bruker, Road Billerica, MA, USA). PHA was chemically extracted and purified prior to NMR analysis. One gram of PHA was extracted using 100 mL of chloroform and precipitated in methanol. Around 10 mg of purified PHA was dissolved in 1 mL of deuterated chloroform and proceeded for NMR analysis at 500 MHz with 64 scans. Tetramethylsilane was used as an internal standard. The spectra for each monomer were recorded and calculated by comparing with a previous publication [7].

### 2.8. Statistical Analysis

All data were collected in triplicate, and statistical results were analyzed using SPSS software. The data comparison was analyzed through a one-way analysis of variance (ANOVA) test with the Tukey test (post hoc test). *p*-values < 0.05 interpreted statistically significant results.

## 3. Results and Discussion

### 3.1. Comparative Analysis of A. vinelandii Wild Type and ∆Avin_16040 Mutant Strains Links PHA Biosynthesis Efficiency to the S-Layer

PHA synthases from both *A. vinelandii* strains were evaluated in terms of expression activity and gene sequences. The PHA synthase activity was analyzed by measuring the release of CoA from 3HB-CoA using a spectrophotometer. According to Table 2, the PHA synthase activities of the wild type and mutant strains were determined to be 395 and 485 U/g of protein, respectively. Previous studies also reported that PHA synthase activities of *Chromobacterium* sp. USM2 and *C*. *necator* harboring the PHA synthase gene from *Aeromonas caviae* were 2462 and 1600 U/g, respectively [26,27]. These synthase activities were much higher than the PHA synthase activities of *A*. *vinelandii*.

Although the cell dry weight (4.9 g/L) and PHA concentration (2.6 g/L) of the mutant strain was higher than that of the wild type strain (3.2 and 1.1 g/L, respectively) (Table 3; no precursor), the activities of synthases in both strains were considerably similar. These results were supported by the number and size of PHA granules in both *A. vinelandii* strains as seen in the transmission electron micrographs (Figure S1). The diameter and number of PHA granules for both *A. vinelandii* strains were comparable, indicating that the PHA synthase activities from both bacterial strains were also similar. Additionally, the molecular weight and polydispersities of both bacterial strains were also similar (Table 4).

According to Sim and colleagues, the molecular weight of PHA is affected by the PHA synthase activity [28]. For more information, both wild type and mutant strains were cultivated under the same conditions including culture medium, food source, cultivation duration, and environment. These results suggested that higher PHA production from the mutant strain compared to the wild type strain was due to the absence of the S-layer. Without the S-layer, the cells could ingest the food source more easily as compared to the wild type strain surrounded by the S-layer.

Since the *A. vinelandii* mutant strain could produce a higher PHA concentration compared to that by the *A. vinelandii* wild type strain, it was essential to compare the PHA synthases that played a role in PHA generation based on their gene sequences. The ability to generate PHA with a different monomer was checked by expressing the gene encoding the PHA synthase from *A. vinelandii* in *C*. *necator* for PHA biosynthesis. Previous studies showed the evaluation of the PHA synthase’s expression in different hosts, and the most frequently employed hosts were *C*. *necator* strains [18,29]. In this study, the PHA synthase of *A. vinelandii* was evaluated by carrying out PHA biosynthesis using *C*. *necator* as a host strain. Prior to that, the gene sequences of the PHA synthase between the wild type and mutant strains of *A. vinelandii* were compared. The only difference between the gene sequences of the two bacterial strains was nucleotide 1166, whereby guanine and adenine were found to be present in the wild type strain and mutant strain, respectively. However, the difference was negligible because the nucleotide sequences for both strains translate to valine (GUG and GUA). Since this study focused on the *A. vinelandii* mutant strain due to the higher PHA accumulation, the PHA synthase from the mutant strain was selected for further analyses.

### 3.2. Identification of Carbon Sources Usable for PHA Production by the PhaC of A. vinelandii in Various Host Strains

Different host strains were used for PHA production in this study. *C. necator* PHB¯4 is a PHA negative mutant that is commonly used for the evaluation of synthases for PHA polymerization from various carbon sources. Meanwhile, *C*. *necator* Re2058 that heterologously expresses a *phaJ* was employed for synthase characterization due to its enhanced ability to accumulate PHA compared to *C. necator* PHB¯4 (Tan et al., 2020, Int. J. Biol. Mol.) and to investigate the possibility of medium-chain-length (mcl) monomer incorporation such as 3-hydroxyhexanoate (3HHx) from oil as a carbon source [18].

Around 4 and 2.1 g/L of dry cell and PHA could be produced by both transconjugated strains using 10 g/L of fructose as a carbon source (Table 5). Similar amounts of PHA (around 2.4 g/L) could be produced by the *A*. *vinelandii* Δ*Avin_16040* mutant strain using 30 g/L of fructose as a carbon source (Table S1). This might be due to competition of carbon source for the production of PHA, alginate, and other components in the *A*. *vinelandii* mutant strain [30]. For *C*. *necator* Re2058 transconjungant, when 10 g/L of CPKO was used as a carbon source, up to 8.6 and 7.7 g/L of dry cell and PHA could be obtained while lesser dry cell and PHA were obtained using *C. necator* PHB¯4 transconjugant. No other mcl monomer was detected, suggesting that this PHA synthase is only able to polymerize PHA with a short-chain-length (scl) monomer.

Glucose, sucrose, and glycerol did not contribute to biomass increment as *C*. *necator* is not able to consume these carbon sources for growth. Molasses, a by-product formed during the sugar production process, was utilized, but only a small increase in biomass was observed. The cells could utilize molasses because molasses was composed of a mixture of monosaccharides, disaccharides, or other complex sugar compounds. Monosaccharides such as fructose in the molasses were consumed, but the amount of fructose might not be sufficient for the enhancement of cell growth and PHA production, as molasses contain other sugars as well. The possibility for *A. vinelandii* to utilize other inexpensive, renewable carbon sources for PHA biosynthesis should be evaluated in future studies, as this not only enables cost-efficient production of PHA, but also serves as a disposal strategy for waste substrates. Soya waste and malt waste have been used by *Alcaligenes latus* as a carbon source to produce P(3HB) [31], showing that renewable resources as such are able to be converted to PHA.

As fructose contributed to better cell growth and PHA accumulation by the transconjugated strains, different concentrations of fructose were also screened to evaluate the best concentration for PHA accumulation (Table S1). Around 20 g/L of fructose yielded the highest PHA amount. The higher fructose concentration did not increase the PHA production but slowly decreased the cell biomass, which might be due to substrate inhibition [32]. After that, a different monomer incorporation was evaluated by adding different precursors during bacterial cultivation. Since the *A*. *vinelandii* wild type strain and *A*. *vinelandii* Δ*Avin_16040* used 30 g/L of fructose for bacterial cell cultivation, the ability of monomer incorporation by transformed strains was also screened using 30 g/L of fructose. Although 20 g/L of fructose yielded the highest PHA amount by the transconjugants, in order to standardize the carbon concentration for the *A*. *vinelandii* wild type strain and *A*. *vinelandii* Δ*Avin_16040* as well as the transconjugants, 30 g/L of fructose was selected to be used as the carbon source for further analysis.

### 3.3. PhaC of A. vinelandii Shows Ability for the Utilization of 4-Hydroxybutyrate

Sodium valerate, sodium 4-hydroxybutyrate, 1,4-butanediol, gamma-butyrolactone, and sodium heptanoate could be utilized for the production of PHA consisting of monomers other than 3HB (Table 3). *A. vinelandii* was known to produce PHA consisting of 3HB and 3HV [11,33]. Interestingly, PHA containing 4HB was found to be produced from structurally related precursors. This is a new finding as this bacterium is not previously known to synthesize PHA containing 4HB. In both native and heterologous host strains, PhaC of *A*. *vinelandii* can synthesize PHA containing 4HB, indicating the inherent ability of the synthase to accommodate and utilize the 4HB substrate.

PHA containing approximately 10 mol% of 4HB was produced by the *A*. *vinelandii* Δ*Avin_16040* mutant strain from 2 g/L of sodium 4HB. PHA that consists of 4HB is known to be degraded by lipase and can be applied in the medical field as a scaffold or other surgical materials. Incorporation of 4HB not only alters the properties of PHA but also increases the value of PHA for application [34]. The wild type strain of *A*. *vinelandii* also could utilize the precursors and produce PHA containing similar composition of 4HB, as shown in Table 3. Previous studies showed that 4HB could be polymerized by several wild type strains including *C*. *necator*, *Delftia acidovorans*, *A*. *latus*, *Rhodococcus ruber*, *Comamonas testosteronii*, and *Hydrogenophaga pseudoflava* [35,36,37,38,39].

The presence of 4HB monomer in the PHA produced by the *A*. *vinelandii* Δ*Avin_16040* mutant strain was further confirmed by ^1^H NMR analysis (Figure S2). Around 9 mol.% of 4HB was measured based on the peaks on the chromatogram, and the results did not deviate much compared to the results obtained from GC analysis (10 mol%). Since there is a lack of publications on the production of PHA with different monomers by *A*. *vinelandii*, there might be unknown inhibition on the PHA biosynthesis pathway by other components in the cell, such as competition of carbon source for both PHA and alginate productions. The metabolic pathway for 4HB production was usually more direct compared to that for 3HB production since 4HB could be formed directly via a reaction between synthase and a structurally related carbon source or precursor, whereas formation of 3HB requires three main reactions through enzyme catalysis [40,41]. Hence, the carbon source used for 3HB production was channeled towards the production of other components, while the precursor was directly used to generate 4HB, thus, increasing the 4HB monomer composition and reducing the 3HB monomer composition.

The substrate specificity of the PHA synthase was further evaluated in *C*. *necator* transconjugants (Table 3). Although more dry cells and PHA could be obtained from the transconjugants as compared to the *A*. *vinelandii* strains, the monomeric composition of produced PHA remains the same. For *A*. *vinelandii* strains, PHA containing up to 10 mol% of 4HB was obtained by utilizing sodium 4-hydroxybutyrate, while *C. necator* transconjugants could only synthesize PHA with a lower 4HB fraction, which was approximately 4–5 mol%. The cell dry weight and PHA content of *C. necator* transconjugants grown on 1,4-butanediol is relatively high, and 4HB fraction is approximately 5 mol%. As discussed previously, a higher 4HB molar fraction in the PHA synthesized by *A*. *vinelandii* strains could be obtained from structurally related precursors such as sodium 4-hydroxybutyrate, as 3HB monomer production is decreased from carbon sources such as fructose, which is partially used for the production of alginate. Meanwhile, *C*. *necator* transconjugants do not possess pathways for alginate production, and since there is no competition for carbon source used for 3HB production, the 4HB fraction in the resultant PHA is comparatively lower. Of notable interest is that PHA with a higher 3HV molar fraction (6 to 15 mol%) could be synthesized by *A*. *vinelandii* strains and *C. necator* PHB^−^4 transconjugants when sodium heptanoate is used as precursor.

Table 6 shows that increasing concentrations of sodium 4-hydroxybutyrate could increase the 4HB monomer content in the PHA produced by the *A*. *vinelandii* Δ*Avin_16040* mutant strain and *C*. *necator* transconjugants. However, the cell dry weight exhibited a significant reduction from 4 to 2 g/L when sodium 4-hydroxybutyrate concentration was increased from 2 to 10 g/L. The reduction in cell dry weight was primarily due to the decrease in PHA concentration since the obtained residual cell biomass was similar regardless of sodium 4-hydroxybutyrate concentration. Toxicity of precursor compounds were previously reported to impact cell growth and PHA accumulation negatively [42]. Although the cell dry weight decreased due to increased precursor concentration, the 4HB molar fraction increased from 10 to 22 mol%. Increments in 4HB molar fraction could change the polymer properties to suit a wider range of applications. Taken together, the data suggest that the PhaC of *A*. *vinelandii* has a preference for 4HB monomer utilization.

## 4. Conclusions

In conclusion, the presence of the S-layer is a key factor affecting PHA biosynthesis in *A. vinelandii* cells. Comparisons between the *A*. *vinelandii* wild type and *A*. *vinelandii* Δ*Avin_16040* mutant strains in terms of gene or amino acid sequences, synthase activity, and granule morphology revealed no significant differences. However, PHA production by the mutant strain without the S-layer surrounding the cell was higher compared to that by the wild type strain. PHA biosynthesis using different carbon sources and precursors revealed the specificity of the PhaC from *A*. *vinelandii* for scl-PHA monomers, making this PhaC a new addition to the group of Class I PHA synthases. An interesting discovery in this study was that the PhaC of *A*. *vinelandii* had the inherent ability to polymerize 4HB, accumulating PHA with 4HB fractions from 10 mol% to as high as 22 mol%. Incorporation of the 4HB monomer into PHA can increase the value of the resultant copolymer, as it is a suitable material in the medical field due to its properties, such as better elasticity and ability to be degraded by lipase. In-depth studies to elucidate the relationship among the S-layer, PHA production, and recovery, as well as alginate production, and efforts to produce PHA with higher fractions of 4HB, could be carried out in the future.

## Figures and Tables

**Table 1:** Strains and plasmids used in this study.

Strain or Plasmid	Description	Reference or Source
***Azotobacter vinelandii* strains**		
ATCC 12837	Wild type strain	Lipman ATCC 12837
Δ*Avin_16040*	Mutant strain derived from wild type 3a strain with *Avin_16040* deletion, Km resistant	[16]
***Cupriavidus necator* strains**		
PHB¯4	PHA-negative mutant of H16	[17]
PHB¯4/*phaC_Av_*	Insertion of PhaC gene from *A*. *vinelandii* mutant strain, made by pBBR1MCS-2	This study
Re2058	H16 Δ*phaC1* and Δ*proC*	[18]
Re2058/*phaC_Av_*	Insertion of PhaC gene from *A*. *vinelandii* mutant strain, made by pCB113	This study
***Escherichia coli* strains**		
*E*. *cloni* 10G	Cloning strain	Lucigen
S17-1	Strain for conjugative transfer of plasmids to *C*. *necator*	[19]
**Plasmid**		
pBBR1MCS-2	Vector for plasmid-based gene expression in *C*. *necator*, Km resistant	[20]
pCB113	pBBR1MCS-2 with the PHA operon from Re2152 (H16 with deletion of *phaB* and *phaC1* and insertion of *phaC2_Ra_*) cloned between *Kpn*I and *Hind*III sites and *C*. *necator proC* region cloned into *Age*I site	[18]

**Table 2:** PHA synthase activities of both *A*. *vinelandii* ATCC 12837 and Δ*Avin_16040* mutant strains.

Bacterial Strain ^a^	PHA Synthase Activity (U/g)
*A*. *vinelandii* ATCC 12837 wild type strain	395
*A*. *vinelandii* Δ*Avin_16040* mutant strain	485

^a^ All bacterial strains were cultivated in MMPHA broth at 30 °C and 200 rpm for 24 h.

**Table 3:** Biosynthesis of different PHAs by *A*. *vinelandii* wild type strain, *A*. *vinelandii* Δ*Avin_16040* mutant strain, *C*. *necator* PHB¯4 containing *phaC* of *A*. *vinelandii* mutant cell and *C*. *necator* Re2058 containing *phaC* of *A*. *vinelandii* mutant cell supplemented with 2 g/L of precursor.

Strain	Precursor ^1^	Cell Dry Weight (g/L) ^2^	PHA Content (%) ^3^	PHA Concentration (g/L) ^4^	Residual Biomass (g/L) ^5^	Monomer Composition (mol %)		
						3HB	3HV	4HB
*A*. *vinelandii* wild type strain	Sodium propionate	6.4 ± 0 ^m^	43 ± 1 ^kl^	2.8 ± 0.1 ^m^	3.6 ± 0 ^s^	100 ± 0	-	-
	Sodium valerate	5.1 ± 0 ^f^	40 ± 0 ^jk^	2.1 ± 0 ^kl^	3.0 ± 0 ^p^	>99 ± 0	<1 ± 0	-
	Sodium 4-hydroxybutyrate	3.2 ± 0.1 ^f^	33 ± 0 ^fgh^	1.0 ± 0 ^def^	2.2 ± 0.1 ^hij^	90 ± 0	-	10 ± 0
	1,4-butanediol	3.2 ± 0.1 ^f^	32 ± 1 ^fgh^	1.0 ± 0 ^def^	2.2 ± 0.1 ^hij^	97 ± 1	-	3 ± 1
	Gamma butyrolactone	2.6 ± 0.1 ^de^	33 ± 0 ^fgh^	0.8 ± 0 ^de^	1.8 ± 0.1 ^fg^	97 ± 0	-	3 ± 0
	Sodium 5-hydroxyvalerate	2.6 ± 0.1 ^jk^	34 ± 1 ^ghi^	1.7 ± 0 ^hij^	1.9 ± 0.1 ^fgh^	100 ± 0	-	-
	4-hydroxyvalerate	0.2 ± 0.1 ^a^	3 ± 1 ^ab^	0^a^	0.2 ± 0.1 ^ab^	100 ± 0	-	-
	Sodium hexanoate	4.3 ± 0.1 ^hi^	34 ± 1 ^gh^	1.4 ± 0 ^gh^	2.9 ± 0.1 ^op^	100 ± 0	-	-
	Sodium heptanoate	4.5 ± 0 ^i^	28 ± 1 ^f^	1.2 ± 0.1 ^fg^	3.3 ± 0 ^qr^	94 ± 0	6 ± 0	-
	Sodium octanoate	2.4 ± 0.1 ^d^	30 ± 2 ^fg^	0.7 ± 0.1 ^cd^	1.7 ± 0.1 ^ef^	100 ± 0	-	-
	Sodium nonanoate	0.1 ± 0 ^a^	N.D.	N.D.	0.1 ± 0 ^a^	N.D.	N.D.	N.D.
	No precursor	3.2 ± 0.1 ^f^	35 ± 1 ^hi^	1.1 ± 0.1 ^ef^	2.1 ± 0.1 ^hij^	100 ± 0	-	-
*A*. *vinelandii* Δ*Avin_16040* mutant strain	Sodium propionate	6.7 ± 0 ^mno^	50 ± 1 ^mn^	3.3 ± 0 ^n^	3.4 ± 0 ^rs^	100 ± 0	-	-
	Sodium valerate	4.3 ± 0 ^hi^	47 ± 2 ^lm^	2.0 ± 0.1 ^jkl^	2.3 ± 0 ^jkl^	99 ± 0	1 ± 0	-
	Sodium 4-hydroxybutyrate	4.0 ± 0 ^gh^	54 ± 1 ^no^	2.2 ± 0 ^l^	1.8 ± 0 ^fg^	90 ± 0	-	10 ± 0
	1,4-butanediol	4.0 ± 0.1 ^gh^	54 ± 1 ^no^	2.1 ± 0 ^l^	1.9 ± 0.1 ^fgh^	97 ± 1	-	3 ± 1
	Gamma butyrolactone	3.7 ± 0 ^g^	50 ± 2 ^mn^	1.8 ± 0.1 ^ijk^	1.9 ± 0 ^fgh^	97 ± 0		3 ± 0
	Sodium 5-hydroxyvalerate	4.1 ± 0 ^i^	52 ± 1 ^mn^	2.1 ± 0 ^l^	2.0 ± 0 ^ghi^	100 ± 0	-	-
	4-hydroxyvalerate	1.7 ± 0.1 ^c^	14 ± 1 ^de^	0.2 ± 0 ^ab^	1.5 ± 0.1 ^de^	100 ± 0	-	-
	Sodium hexanoate	5.3 ± 0.1 ^k^	52 ± 1 ^mno^	2.7 ± 0.1 ^m^	2.6 ± 0.1 ^mn^	100 ± 0	-	-
	Sodium heptanoate	4.5 ± 0 ^i^	31 ± 0 ^hij^	1.4 ± 0 ^hi^	3.1 ± 0 ^pq^	88 ± 0	12 ± 0	-
	Sodium octanoate	2.8 ± 0.1 ^e^	15 ± 4 ^de^	0.4 ± 0.1 ^b^	2.4 ± 0.1 ^klm^	100 ± 0	-	-
	Sodium nonanoate	<0.1 ± 0 ^a^	N.D.	N.D.	<0.1 ± 0 ^a^	N.D.	N.D.	N.D.
	No precursor	4.9 ± 0.1 ^j^	53 ± 3 ^no^	2.6 ± 0.2 ^m^	2.3 ± 0.1 ^jkl^	100 ± 0	-	-
*C*. *necator* PHB¯4 containing *phaC* of *A*. *vinelandii* mutant cell	Sodium propionate	4.4 ± 0 ^i^	39 ± 1 ^ijk^	1.7 ± 0 ^hij^	2.7 ± 0 ^no^	99 ± 0	1 ± 0	-
	Sodium valerate	6.7 ± 0 ^mno^	7 ± 0 ^bc^	0.5 ± 0 ^bc^	6.2 ± 0 ^t^	98 ± 1	2 ± 1	-
	Sodium 4HB	6.5 ± 0.1 ^mn^	66 ± 1 ^q^	4.3 ± 0.1 ^o^	2.2 ± 0.1 ^ijk^	95 ± 1	-	5 ± 1
	1,4-butanediol	7.0 ± 0 ^op^	80 ± 1 ^tuv^	5.6 ± 0.1 ^q^	1.4 ± 0 ^d^	96 ± 0	-	4 ± 0
	Gamma butyrolactone	6.7 ± 0 ^mno^	72 ± 0 ^rs^	4.8 ± 0 ^p^	1.9 ± 0 ^fgh^	95 ± 0		5 ± 0
	Sodium 5-hydroxyvalerate	5.7 ± 0 ^l^	55 ± 1 ^o^	3.2 ± 0.1 ^n^	2.5 ± 0 ^lmn^	100 ± 0	-	-
	4-hydroxyvalerate	<0.1 ± 0 ^a^	N.D.	N.D.	<0.1 ± 0 ^a^	N.D.	N.D.	N.D.
	Sodium hexanoate	0.2 ± 0 ^a^	11 ± 1 ^cd^	<0.1 ± 0 ^a^	0.1 ± 0 ^ab^	100 ± 0	-	-
	Sodium heptanoate	1.3 ± 0.2 ^b^	18 ± 1 ^e^	0.2 ± 0 ^ab^	1.1 ± 0.2 ^c^	85 ± 1	15 ±1	-
	Sodium octanoate	0.1 ± 0 ^a^	N.D.	N.D.	0.1 ± 0 ^ab^	N.D.	N.D.	N.D.
	Sodium nonanoate	0.2 ± 0 ^a^	N.D.	N.D.	0.2 ± 0 ^ab^	N.D.	N.D.	N.D.
	No precursor	7.4 ± 0 ^q^	61 ± 1 ^p^	4.5 ± 0 ^o^	2.9 ± 0 ^op^	100 ± 0	-	-
*C*. *necator* Re2058 containing *phaC* of *A*. *vinelandii* mutant cell	Sodium propionate	7.1 ± 0 ^pq^	70 ± 1 ^qr^	5.0 ± 0.1 ^p^	2.1 ± 0 ^hij^	100 ± 0	-	-
	Sodium valerate	8.4 ± 0 ^r^	79 ± 1 ^tu^	6.6 ± 0 ^s^	1.8 ± 0 ^fg^	99 ± 1	1 ± 1	-
	Sodium 4HB	8.8 ± 0 ^s^	76 ± 2 ^st^	6.7 ± 0.2 ^s^	2.1 ± 0 ^hij^	94 ± 0	-	6 ± 0
	1,4-butanediol	10.4 ± 0.1 ^u^	83 ± 2 ^uv^	8.6 ± 0.2 ^u^	1.8 ± 0.1 ^fg^	95 ± 0	-	5 ± 0
	Gamma butyrolactone	7.1 ± 0 ^pq^	85 ± 3 ^v^	6.0 ± 0.2 ^r^	1.1 ± 0 ^c^	95 ± 1		5 ± 1
	Sodium 5-hydroxyvalerate	6.8 ± 0.2 ^nop^	66 ± 1 ^pq^	4.5 ± 0.1 ^o^	2.3 ± 0.2 ^jkl^	100 ± 0	-	-
	4-hydroxyvalerate	<0.1 ± 0 ^a^	N.D.	N.D.	<0.1 ± 0 ^a^	N.D.	N.D.	N.D.
	Sodium hexanoate	9.2 ± 0.2 ^t^	81 ± 1 ^tuv^	7.4 ± 0.1 ^t^	1.8 ± 0.2 ^fg^	100 ± 0	-	-
	Sodium heptanoate	8.4 ± 0 ^r^	77 ± 1 ^st^	6.5 ± 0.1 ^s^	1.9 ± 0 ^fgh^	99 ± 1	1 ±1	-
	Sodium octanoate	0.3 ± 0 ^a^	N.D.	N.D.	0.3 ± 0 ^b^	N.D.	N.D.	N.D.
	Sodium nonanoate	0.2 ± 0 ^a^	N.D.	N.D.	0.2 ± 0 ^ab^	N.D.	N.D.	N.D.
	No precursor	11.0 ± 0 ^v^	83 ± 1 ^uv^	9.1 ± 0.1 ^v^	1.9 ± 0 ^fgh^	100 ± 0	-	-

Data shown are means of triplicate. The superscripts represent the significant difference of the data using statistical analysis (*p* < 0.05). Superscript alphabets that are different indicate significant difference. ^1^ Cells were cultivated in MMPHA at 30 °C, 200 rpm with 30 g/L of fructose as carbon source and 0.54 g/L of urea as nitrogen source. All strains were cultivated for 72 h. ^2^ Cell dry weight was obtained after freeze-drying process. ^3^ PHA content of freeze-dried cell was determined using gas chromatography. ^4^ PHA concentration = cell dry weight * (PHA content/100). ^5^ Residual biomass = cell dry weight − P(3HB) concentration.

**Table 4:** Characteristics of P(3HB) produced by *A. vinelandii* ATCC 12837 wild type and ∆*Avin_16040* mutant strains.

Strain to Produce P(3HB)	*M*_w_ (×10^6^ Da)	*M*_n_ (×10^6^ Da)	Ð	*T*_m_ (°C)	*T*_c_ (°C)	*T*_g_ (°C)	Tensile Strength (MPa)	Elongation at Break (%)	Young’s Modulus (GPa)	References
*A. vinelandii* ATCC 12837 wild type strain	1.7	0.6	3.0	173	44	−1	35	9.6	2.1	This study
*A. vinelandii* ∆*Avin_16040* mutant strain	1.9	0.7	2.8	172	42	−7	37	7.8	3.7	This study
*C*. *necator*	0.01–3	0.2–0.7	1.7–2.9	180	-	4	43	5	3.5	Doi, 1990

**Table 5:** Biosynthesis of PHAs by *C*. *necator* PHB^−^4 and *C*. *necator* Re2058 harbouring *phaC* of *A*. *vinelandii* mutant strain using different carbon sources.

Strain				*A*. *vinelandii* Δ*Avin_16040* Mutant Strain		*C*. *necator* PHB^−^4		*C*. *necator* Re2058	
Carbon Source ^1^	Cell Dry Weight (g/L) ^2^	PHA Content (%) ^3^	PHA Concentration (g/L) ^4^	Cell Dry Weight (g/L) ^2^	PHA Content (%) ^3^	PHA Concentration (g/L) ^4^	Cell Dry Weight (g/L) ^2^	PHA Content (%) ^3^	PHA Concentration (g/L) ^4^
Glucose	3.7 ± 0.1 ^e^	42 ± 1 ^c^	1.6 ± 0 ^c^	0.1 ± 0 ^a^	2 ± 1 ^a^	0 ^a^	0.1 ± 0 ^a^	1 ± 0 ^a^	0 ^a^
Fructose	3.7 ± 0.1 ^e^	50 ± 3 ^d^	1.9 ± 0.1 ^d^	4.1 ± 0.2 ^d^	51 ± 1 ^b^	2.1 ± 0.1 ^b^	4.0 ± 0 ^c^	51 ± 2 ^b^	2.0 ± 0.1 ^b^
Sucrose	3.3 ± 0 ^d^	40 ± 2 ^c^	1.3 ± 0.1 ^b^	0.3 ± 0 ^a^	4 ± 0 ^a^	0 ^a^	0.4 ± 0 ^a^	5 ± 0 ^a^	0 ^a^
CPKO	<0.1 ± 0 ^a^	N.D.	N.D.	3.5 ± 0.2 ^c^	54 ± 2 ^c^	1.9 ± 0.2 ^b^	8.6 ± 0.3 ^d^	89 ± 2 ^c^	7.7 ± 0.4 ^c^
Molasses	3.1 ± 0.1 ^c^	23 ± 0 ^a^	0.7 ± 0 ^a^	1.2 ± 0.1 ^b^	2 ± 0 ^a^	0 ^a^	1.2 ± 0 ^b^	4 ± 0 ^a^	0 ^a^
Glycerol	2.5 ± 0 ^b^	29 ± 0 ^b^	0.7 ± 0 ^a^	0.4 ± 0.1 ^a^	4 ± 0 ^a^	0 ^a^	0.3 ± 0 ^a^	1 ± 0 ^a^	0 ^a^

Data shown are means of triplicate. The superscripts represent the significant difference of the data using statistical analysis (*p* < 0.05). Superscript alphabets for each column that are different indicate a significant difference. ^1^ Cells were cultivated in MMPHA at 30 °C, 200 rpm for 48 h with 0.54 g/L of urea as nitrogen source. 20 g/L of carbon source was used for *A*. *vinelandii* Δ*Avin_16040* mutant strain while 10 g/L of carbon source was used for *C*. *necator* transconjugants. ^2^ Cell dry weight was obtained after freeze-drying process. ^3^ PHA content of freeze-dried cell was determined using gas chromatography. ^4^ PHA concentration = cell dry weight * (PHA content/100).

**Table 6:** Cell dry weight, PHA production, and monomer composition of PHA produced by *A*. *vinelandii* Δ*Avin_16040* mutant strain, *C*. *necator* PHB^−^4, and *C*. *necator* Re2058 transconjugants.

Strain ^1^	Concentrations of Na4HB (g/L)	Cell Dry Weight (g/L) ^2^	PHA Content (%) ^3^	PHA Concentration (g/L) ^4^	Monomer Composition (mol.%)	
					3HB	4HB
*A*. *vinelandii* Δ*Avin_16040* mutant strain	2	4.0 ± 0 ^e^	54 ± 1 ^d^	2.2 ± 0 ^e^	90 ± 0	10 ± 0
	4	3.5 ± 0 ^d^	48 ± 0 ^c^	1.7 ± 0 ^d^	87 ± 0	13 ± 0
	6	3.0 ± 0.1 ^c^	42 ± 1 ^bc^	1.3 ± 0 ^c^	84 ± 0	16 ± 0
	8	2.5 ± 0.1 ^b^	36 ± 0 ^ab^	0.9 ± 0 ^b^	80 ± 0	20 ± 0
	10	2.0 ± 0.1 ^a^	34 ± 3 ^a^	0.7 ± 0 ^a^	78 ± 0	22 ± 0
	No precursor	4.3 ± 0.1 ^f^	55 ± 2 ^d^	2.4 ± 0 ^f^	100 ± 0	0
*C*. *necator* PHB^−^4 containing *phaC* of *A*. *vinelandii* mutant cell	0.5	3.7 ± 0 ^a^	40 ± 3 ^a^	1.5 ± 0.1 ^a^	95 ± 0	5 ± 0
	1	3.8 ± 0 ^ab^	43 ± 2 ^ab^	1.6 ± 0.1 ^ab^	94 ± 0	6 ± 0
	1.5	4.0 ± 0 ^bc^	46 ± 2 ^ab^	1.8 ± 0.1 ^ab^	93 ± 0	7 ± 0
	2	4.0 ± 0 ^c^	49 ± 3 ^b^	2.0 ± 0.1 ^b^	93 ± 0	7 ± 0
	2.5	4.0 ± 0 ^bc^	45 ± 1 ^ab^	1.8 ± 0 ^ab^	93 ± 0	7 ± 0
	No precursor	4.9 ± 0.1 ^d^	62 ± 2 ^c^	3.0 ± 0.2 ^c^	100 ± 0	0
*C*. *necator* Re2058 containing *phaC* of *A*. *vinelandii* mutant cell	0.5	4.3 ± 0.1 ^b^	45 ± 2 ^a^	2.0 ± 0.1 ^a^	100 ± 0	0
	1	4.6 ± 0 ^c^	49 ± 0 ^b^	2.3 ± 0 ^b^	97 ± 0	3 ± 0
	1.5	4.7 ± 0 ^cd^	50 ± 1 ^b^	2.3 ± 0.1 ^bc^	97 ± 0	3 ± 0
	2	4.8 ± 0 ^de^	51 ± 0 ^b^	2.5 ± 0 ^cd^	96 ± 0	4 ± 0
	2.5	5.0 ± 0 ^e^	53 ± 1 ^b^	2.6 ± 0.1 ^d^	95 ± 0	5 ± 0
	No precursor	4.0 ± 0 ^a^	45 ± 1 ^a^	1.8 ± 0 ^a^	100 ± 0	0

Data shown are means of triplicate. The superscripts represent the significant difference of the data using statistical analysis (*p* < 0.05) for each bacterial strain. Superscript alphabets for each column that are different indicate a significant difference.^1^ *A*. *vinelandii* Δ*Avin_16040* mutant strain and the transconjugants were cultivated using 30 g/L of fructose and 0.54 g/L of urea at 30 °C with agitation speed of 200 rpm. *A*. *vinelandii* Δ*Avin_16040* mutant strain was cultivated for 72 h while the transconjugants were cultivated for 48 h. Different concentrations of Na4HB were used. ‘No precursor’ indicates no addition of precursor in the medium. ^2^ Cell dry weight was obtained after freeze-drying process. ^3^ PHA content of freeze-dried cell was determined using gas chromatography. ^4^ PHA concentration = cell dry weight * (PHA content/100).

## Data Availability

Not applicable.

## Supplementary Materials

The following are available online at https://www.mdpi.com/article/10.3390/polym13101576/s1, Figure S1: TEM micrographs *A. vinelandii* ATCC 12837 wild type strain and *A. vinelandii* ∆*Avin_16040* mutant strain. Figure S2: ^1^H NMR spectrum of P(3HB-*co*-10 mol% 4HB) produced by *A*. *vinelandii* Δ*Avin_16040*. Table S1. Biosynthesis of P(3HB) by *A*. *vinelandii* Δ*Avin_16040* mutant strain, *C*. *necator* PHB¯4, and *C*. *necator* Re2058 harbouring *phaC* of *A*. *vinelandii* mutant cell using different concentrations of fructose.
