# Supplementary material for: In Vivo Characterization and Application of the PHA Synthase from Azotobacter vinelandii for the Biosynthesis of Polyhydroxyalkanoate Containing 4-Hydroxybutyrate
Source: Polymers (Basel). 2021 May 14;13(10):1576. doi: 10.3390/polym13101576 (PMC8156725; doi:10.3390/polym13101576)
Supplement: Supplementary file 1 [file polymers-13-01576-s001.zip › polymers-1213858-supplementary.pdf]

Supplementary Materials

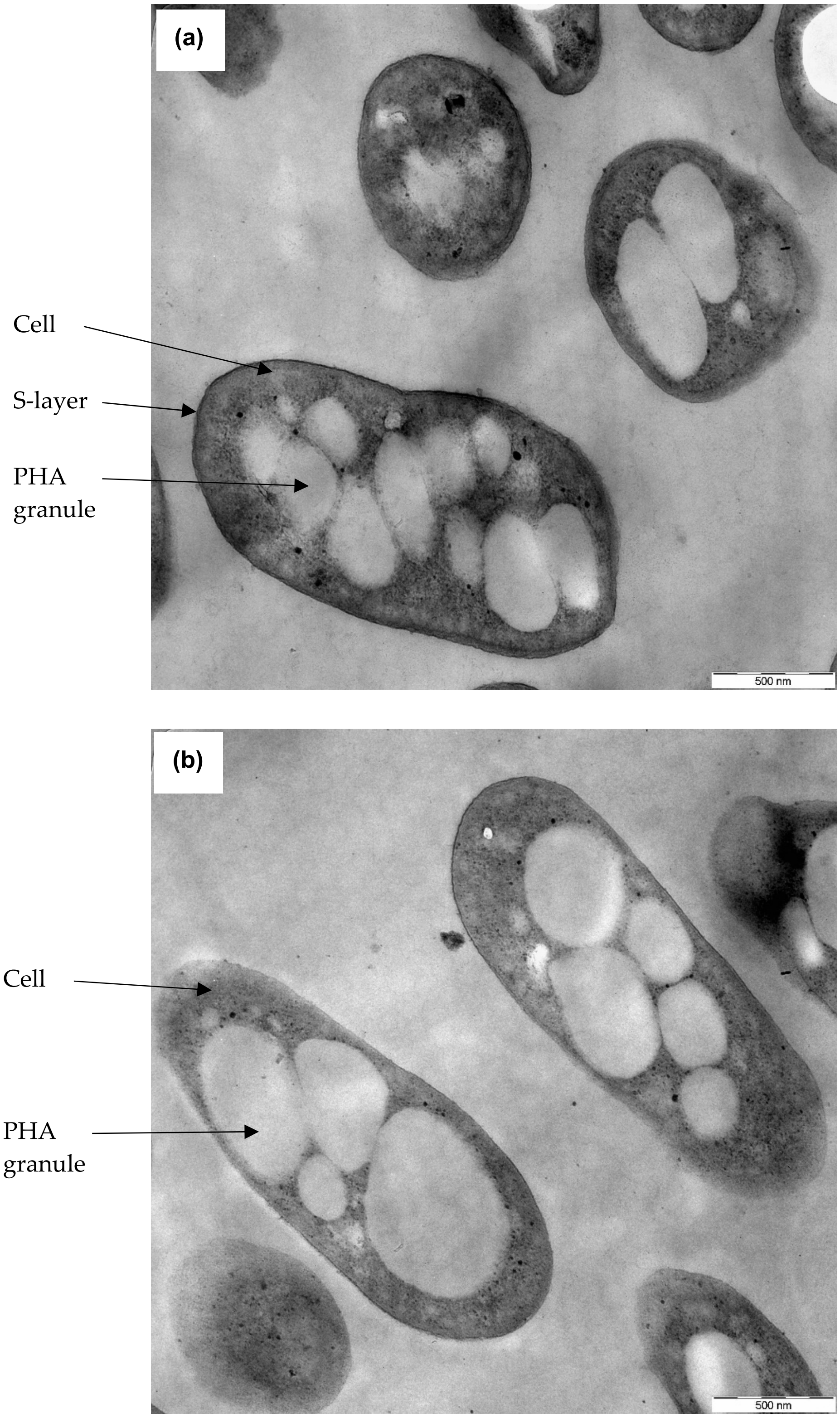

Figure S1. TEM micrographs *A. vinelandii* ATCC 12837 wild type strain and *A. vinelandii* Δ*Avin_16040* mutant strain. (**a**) *A. vinelandii* ATCC 12837 wild type strain and (**b**) *A. vinelandii* Δ*Avin_16040* mutant strain.

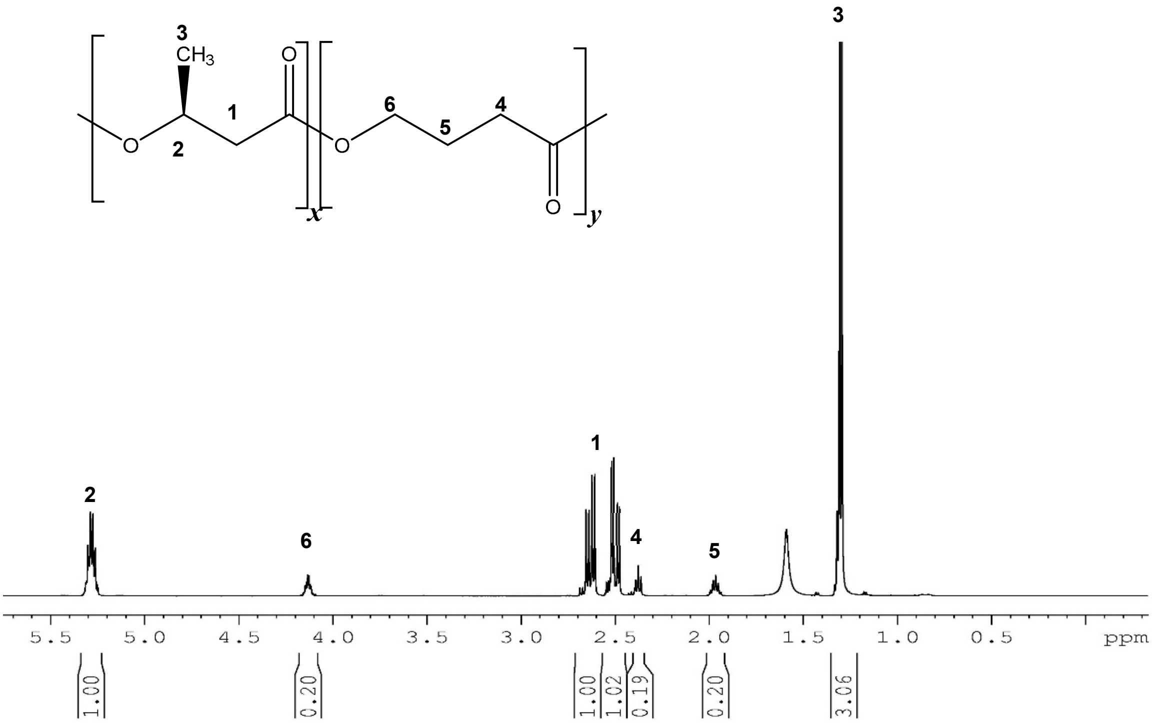

Figure S2: ^{1}H NMR spectrum of P(3HB-*co*-10 mol% 4HB) produced by *A. vinelandii* Δ*Avin_16040*. The composition of the polymer was determined by 500 MHz ^{1}H NMR spectra. Tetramethylsilane (Me_4Si) was used as an internal chemical shift standard.

Table S1. Biosynthesis of P(3HB) by *A. vinelandii* Δ*Avin_16040* mutant strain, *C. necator* PHB$^-$4, and *C. necator* Re2058 harbouring *phaC* of *A. vinelandii* mutant cell using different concentrations of fructose.

Strain	Concentration of Fructose (g/L) [1]	Cell Dry Weight (g/L) [2]	P(3HB) Content (%) [3]	P(3HB) Concentration (g/L) [4]
A. vinelandii Δ*Avin_16040* mutant strain	10	1.6 ± 0 [a]	16 ± 1 [a]	0.3 ± 0 [a]
	20	3.6 ± 0.2 [b]	55 ± 2 [b]	2.0 ± 0.1 [b]
	30	4.2 ± 0.1 [c]	58 ± 1 [bc]	2.4 ± 0.1 [c]
	40	4.2 ± 0.1 [c]	63 ± 2 [c]	2.7 ± 0.1 [c]
	50	4.4 ± 0.2 [c]	60 ± 3 [bc]	2.6 ± 0.2 [c]
C. necator PHB$^-$4	10	3.7 ± 0 [a]	43 ± 0 [a]	1.6 ± 0 [a]
	20	7.3 ± 0.1 [d]	77 ± 1 [c]	5.6 ± 0.1 [c]
	30	7.5 ± 0 [d]	77 ± 1 [c]	5.8 ± 0.1 [c]
	40	6.9 ± 0 [c]	84 ± 1 [d]	5.8 ± 0.1 [c]
	50	5.7 ± 0.1 [b]	73 ± 1 [b]	4.1 ± 0.1 [b]
C. necator Re2058	10	4.1 ± 0 [a]	50 ± 1 [a]	2.0 ± 0 [a]
	20	7.6 ± 0 [d]	77 ± 1 [c]	5.9 ± 0.1 [e]
	30	7.4 ± 0.1 [c]	74 ± 1 [b]	5.4 ± 0 [d]
	40	7.1 ± 0.1 [c]	73 ± 1 [b]	5.2 ± 0 [c]
	50	6.9 ± 0 [b]	73 ± 1 [b]	5.0 ± 0.1 [b]

Data shown are means of triplicate. The superscripts represent the significant difference of the data using statistical analysis ($P < 0.05$). Superscript alphabets in each column for each bacterial strain that are different indicate a significant difference.

[1] Cells were cultivated in MMPHA at 30 °C, 200 rpm for 48 h with different concentrations of fructose and 0.54 g/L of urea as nitrogen source.

[2] Cell dry weight was obtained after freeze-drying process.

[3] P(3HB) content of freeze-dried cells was determined using gas chromatography.

[4] P(3HB) concentration = cell dry weight * [P(3HB) content/100].
